# Effect of delayed ICU admission on mortality and morbidity

**DOI:** 10.1186/cc9878

**Published:** 2011-03-11

**Authors:** R Ramaiah, B Shepard, P Hopkins, R Maharaj

**Affiliations:** 1 King's College Hospital, London, UK

## Introduction

Delayed admissions to the ICU from the Emergency Department (ED) may be associated with increase in mortality and morbidity [1]. We wanted to answer the following questions: is there an association between the timing of presentation to the ED and mortality; and is the time interval between the patient presenting to the ED and admission to the ICU associated with increase in mortality and morbidity?

## Methods

We collected the number of patients admitted from the ED to the ICU from April 2009 to March 2010. The time duration from the patient presenting to the ED and the patient admitted to the ICU was collected. We defined any admission to the ICU more than 4 hours from the ED as delayed admission, as per the national standards. We assessed the APACHE score of the patient on admission, the length of stay in the ICU (LOS), relationship to the time of ED admission (either office hours 08:00 to 17:00 or out of hours 17:01 to 07:59) and the hospital mortality associated with each admission.

## Results

We had 547 admissions to the ICU from the ED. There was no significant association between out of office admission to the ED and hospital mortality (OR = 0.858, 95% CI = 0.457 to 1.610) after adjustment for age and APACHE score. There was also no statistically significant difference between patients that took more than 4 hours between ED and ICU, with respect to the hospital mortality (OR = 1.00 and 95% CI = 0.999 to 1.001). We performed a COX regression analysis to establish whether delays were associated with increased LOS, using age and APACHE as the covariates. There was no statistically significant association between ICU LOS and delays to ICU admission (hazard ratio = 0.948 and 95% CI = 0.934 to 0.962). There was no difference between APACHE scores >25 and ICU admission delays (chi-square *P *= 0.897).

## Conclusions

There was no association between delay in ED to ICU admission on mortality or length of stay in the hospital. This might be due to the fact that the sick patients presenting in the ED are seen by a physician early, thereby leading to appropriate triage of the patient to the ICU. APACHE II scoring seems to be an independent variable and has a linear relation to the mortality and length of stay in the hospital.
